# Personality psychopathology, drug use and psychological symptoms in adolescents with substance use disorders and community controls

**DOI:** 10.7717/peerj.992

**Published:** 2015-06-09

**Authors:** Ernesto Magallón-Neri, Rosa Díaz, Maria Forns, Javier Goti, Josefina Castro-Fornieles

**Affiliations:** 1 Department of Child and Adolescent Psychiatry and Psychology, Institute of Neurosciences Hospital Clinic Universitari of Barcelona, Spain; 2 Department of Personality, Assessment and Psychological Treatment, Faculty of Psychology, University of Barcelona, Spain; 3 Institute of Research in Brain, Cognition and Behavior (IR3c), Spain; 4 IDIBAPS (Institut d’Investigacions Biomediques August Pi Sunyer), Barcelona, Spain; 5 Department of Psychiatry and Clinical Psychobiology, University of Barcelona, Spain

**Keywords:** Personality psychopathology five (PSY-5), Dimensional, Substance use disorders, Internalizing–externalizing symptoms, Adolescents

## Abstract

Substance use is a risk behavior that tends to increase during adolescence, a time when part of the personality is still in development. Traditionally, personality psychopathology has been measured in terms of categories, although dimensional models have demonstrated better consistency. This study aimed to analyze differences in personality profiles between adolescents with substance use disorders (SUD *n* = 74) and matched community controls (MCC *n* = 74) using the Personality Psychopathology Five (PSY-5) dimensional model. Additionally, we compared age at first drug use, level of drug use and internalizing and externalizing symptoms between the groups. In this study, the PSY-5 model has proved to be useful for differentiating specific personality disturbances in adolescents with SUD and community adolescents. The Disconstraint scale was particularly useful for discriminating adolescents with substance use problems and the Delinquent Attitudes facet offered the best differentiation.

## Introduction

Substance use disorders (SUD) are complex entities with multiple biological and environmental risk factors and a wide range of clinical expressions (Hicks, Iacono & McGue, 2014). Their study requires detailed evaluation across several dimensions of substance use and related problems, in addition to an assessment of quantity and frequency of drug use (Kaminer, 2008). One of these dimensions is psychopathological personality traits. Many studies have identified behavioral disinhibition and emotional dysregulation as important factors in the etiology of SUD (Baker et al., 2004; Iacono, Malone & McGue, 2008; Tarter et al., 2003), and different personality traits predict distinct patterns of substance use (Castellanos-Ryan, O’Leary-Barrett & Conrod, 2013; Elkins et al., 2006). The links between personality dimensions and substance behavior appear to be mediated by different reinforcement processes (Woicik et al., 2009). Evidence demonstrates that personality traits can be used as endophenotypes of the risk for SUD, building a bridge between genes and SUD, allowing better understanding of which individual differences provide vulnerability (Belcher et al., 2014).

The Personality Psychopathology Five (PSY-5) is a descriptive, dimensional model of personality. It was initially developed with the Minnesota Multiphasic Personality Inventory-2 (MMPI-2) by Harkness (1992), to complement the categorical diagnosis of personality disorders in adults, and was later adapted for use with adolescents (MMPI-A; McNulty et al., 1997). The model comprises five broad dimensional scales: Aggressiveness, Psychoticism, Disconstraint, Introversion, and Neuroticism or Negative Emotionality (Harkness et al., 2012).

The Aggressiveness scale relates to anger and rage (Harkness, 2009) and aggression used to accomplish goals or intimidate others. High scores on this scale are associated with antagonism, dominance, aggressive tendencies, and ambition, as well as with externalizing subtypes of post-traumatic stress disorder (i.e., Miller et al., 2004). The Aggressiveness scale has two facets: Hostility and Grandiosity/Indignation. The Psychoticism scale measures disconnection from reality with alienation and distrustfulness, and high scores are significant predictors of schizotypal or borderline symptoms with a history of suicide attempts. The Psychoticism scale contains two facets: Psychotic Beliefs/Experiences and Odd Mentation. The Disconstraint scale assesses the extent to which behavior is constrained by consideration of future consequences (Harkness, 2009), and high scores correlate with other personality characteristics such as impulsivity, sensation seeking, and boredom proneness (Harkness et al., 2012). The Disconstraint scale comprises two facets: Delinquent Attitudes and Norm Violation. The Introversion scale, measures the extent to which respondents are inward-looking and focused on internal thoughts, feelings, and moods. High scores are related to low sociability, low energy, and low positive emotions. The Introversion scale is composed by the facets of Low Drive/Expectations and Low Sociability. Finally, the Negative Emotionality scale is considered as a single facet and refers to the sensitivity of the danger detection system and anxiety (i.e., to danger cues). It correlates with harm avoidance, behavioral inhibition, and emotional instability (Harkness et al., 2012). These neurotic personality traits have shown be linked to drinking behavior through a negative reinforcement processes, and linked to the ability of some substances to relieve negative affective states (Woicik et al., 2009).

Most literature on the PSY-5 model has concerned adult populations, and relatively few studies have been conducted with adolescents. Among those that have been conducted with adolescents, the study by McNulty et al. (1997) is noteworthy for describing the adaptation of scales to the adolescent population based on the MMPI-A. Bolinskey et al. (2004) subsequently developed the specific facets for the PSY-5 scales. More recent studies include those by Veltri et al. (2009) and Veltri et al. (2014) with forensic adolescent samples, which found that the Disconstraint scale is a marker of behavioral disinhibition and impulsivity and is associated with nonviolent delinquency, whereas Aggressiveness is characterized by the use of instrumental aggression and interpersonal dominance. In a general adolescent clinical population, Stokes et al. (2009) provided support for the predictive validity of the MMPI-A PSY-5 facet scales, finding that most demonstrate good to excellent internal consistency. Moreover, found that externalizing problems were related most strongly and consistently to the Aggressiveness and the Disconstraint scales, and internalizing problems and symptoms were related to the Negative Emotionality and the Introversion scales. However, not all PSY-5 scales have been fully studied and their usefulness in community control and SUD adolescents has not been compared.

The Disconstraint and Negative Emotionality from the PSY-5 scales seem to capture aspects that are important in the etiology of addictive behaviors (Elkins et al., 2006). High scores on the Disconstraint scale are indicative of behavioral under-control or neurobehavioral disinhibition, which are associated with both earlier onset of heavy substance use and a greater persistence of alcohol use and abuse (Chassin, Pitts & Prost, 2002), and prospectively predict substance-related disorders (Caspi et al., 1996). Tarter et al. (2003) also indicated that neurobehavioral disinhibition is a component of the liability to early age at onset of SUD. On the other hand, high scores on the Negative Emotionality scale are associated with the maintenance of SUD through negative reinforcement (Baker et al., 2004).

In addition to personality characteristics, several emotional and behavioral problems are also commonly associated with substance use in adolescence. Externalizing problems at an early age are associated with later substance use; these problems regularly precede adolescent substance use in both genders. In women, adolescent cannabis and alcohol use predict internalizing disorders in adulthood (Miettunen et al., 2014). Behavioral disinhibition is associated with a higher familial loading, earlier age of initiation, adolescent onset of SUD and a more severe and persistent course of SUD in adulthood (Hicks, Iacono & McGue, 2014). Although the associations are weaker and less consistent, there is also evidence of an internalizing pathway to SUD (Hussong et al., 2011). In adolescents this liability is expressed through a lack of approach and exploratory behavior, passivity, discomfort, shyness and anxiety (Hicks, Iacono & McGue, 2014).

The primary aim of this study was to examine the association between PSY-5 scales (and their facets) and drug use patterns (age at onset and level of drug use) in a group of adolescents with SUD and matched community controls (MCC). The secondary aim was to compare the PSY-5 scales (adolescent report) with the broadband scales of internalizing and externalizing symptoms from the Child Behavior Checklist (CBCL; parents report) in both groups.

The study tested the following hypotheses. First, the PSY-5 scales, especially Disconstraint, would be able to differentiate substance use patterns between SUD cases and MCC. Second, SUD cases would score higher on the Disconstraint and Negative Emotionality scales than community adolescents. Third, early age at onset of drug use would be associated with higher scores on the Disconstraint scale. Fourth, the PSY-5 scales, Negative Emotionality, would be associated with mixed internalizing/externalizing symptoms, Introversion with internalizing symptoms, and Aggressiveness or Disconstraint with externalizing symptoms from the CBCL.

## Method

### Participants

#### Substance use disorder (SUD) group

The study was conducted in a Child and Adolescent Psychiatry Department of a tertiary hospital in Spain. One hundred and twelve patients were referred for treatment for SUD to the Addictive Behaviors Unit of the Child and Adolescent Psychiatry and Psychology Service of a public hospital, of whom 93 (83%) agreed to participate. Among these, 11 did not complete the assessment protocol and 9 were younger than the control group age range. This left a final group of 74 adolescents aged 15–18 years who met the criteria for SUD (for alcohol, cannabis or other drugs excluding tobacco) as defined in the Diagnostic and Statistical Manual of Mental Disorders, Fourth Edition Text Revision (DSM-IV-TR; American Psychiatric Association, 2002). Patients were evaluated by two professionals (a clinical psychologist and a psychiatrist) who both have extensive clinical experience and are specialists in dual disorders in adolescents.

#### Matched community control (MCC) group

We tried to match the SUD group, according to gender, age and academic level, with 74 adolescents from five secondary schools, with no prior psychological or psychiatric diagnosis or treatment. Finally, control subjects were matched according to the gender, taking into account a window of age between +/−6 months with respect to the corresponding SUD subject. The participating schools were selected by means of stratified random sampling in the metropolitan area of Barcelona (divided into five areas according to zip code). Of an initial group of 425 adolescents within the same SUD patients age range (aged 15–18 years), 247 did not provide informed consent to participate in the study, a further 17 failed to attend on the day of assessment, and 44 either did not complete the assessment protocol or reported a history of psychological or psychiatric treatment. From the remaining pool of 117 controls who did complete the protocol, a final group of 74 subjects was matched with the SUD cases. The procedure for discarding subjects with previous psychological treatment was as follows: first, professors were asked orally to preselect subjects who, in their view, did not present mental problems. Participants (and parents) were then asked in a self-report format whether they (or their children) had been previously treated for, or diagnosed with, psychological or psychiatric disorders. Subjects who answered affirmatively were excluded from the analysis.

### Measures

#### Sociodemographic data

A short screening questionnaire was applied to the MCC group in order to obtain data on age, gender, schooling, and so on. In the case of SUD patients, these data were obtained from clinical records.

#### Psychiatric diagnosis

Both SUD and other clinical diagnoses were extracted from the hospital data processing system and verified personally with the clinicians who also reported any changes in the patient’s status. Initial diagnoses of the clinical sample were based on the Spanish version of the Kiddie-SADS semi-structured diagnostic interview for children and adolescents. This instrument has shown good reliability and validity for present and life-time disorders.

#### Substance use

Information regarding age at onset of drug use and the level of use, in terms of the quantity/frequency of the different drugs used, was gathered through semi-structured interviews adapted to Spanish (Díaz et al., 2008) from those used in the Collaborative Study on Genetics of Alcoholism (Hesselbrock et al., 1999).

The level of use of each drug (tobacco, alcohol, cannabis, cocaine, amphetamine derivatives, etc.) was coded by the clinician according to pattern of frequency and characteristics of use into three ordinal categories: (1) No use; (2) Substance use without problems (i.e., drug use at parties, during holidays, or at special celebrations, with no evidence of drug-related problems); and (3) Risky substance use, defined as a quantity-frequency and/or situational pattern of drug use that could lead to health or psychosocial problems in adolescents. This category includes diagnoses of drug abuse or dependence, only presents in the SUD patients group. In the MCC group only occasional alcohol intoxication at parties, more than 4 alcohol units per occasion or weekend use of cannabis were accepted; other patterns of use were considered as exclusion criteria. Participants were classified by the clinical staff according to their reported substance use patterns. In doubtful cases, a consensus was obtained among several clinicians.

#### Personality psychopathology

The Spanish version of the MMPI-A, which shows acceptable psychometric properties (Jiménez-Gómez & Ávila-Espada, 2003), was administered. This self-report contains 478 items assessing personality characteristics and psychopathological symptoms in adolescents. The PSY-5 scales were extracted through an algorithm based on item configuration obtained from the model adapted by Bolinskey et al. (2004). This model contains five broad clinical scales that are reorganized into facets that help to identify those content areas responsible for clinical elevations on the corresponding PSY-5 scale. The model shows good psychometric properties, with a moderate to high index of reliability (Cronbach’s alpha from 0.79 to 0.83 for the domains, and from 0.57 to 0.78 for the facets) (Bolinskey et al., 2004; Archer, 2005). For the present study, the Cronbach’s alphas were from 0.74 to 0.84 for the domains and from 0.52 to 0.80 for the facets. The inter-correlations among PSY-5 scales in the present study ranged between .09 (Introversion-Aggressiveness) to .54 (Disconstraint-Aggressiveness).

#### Behavioral and emotional symptoms

A Spanish version of the original Child Behavior Checklist (CBCL) (Achenbach, 1991) was completed by parents to assess behavioral symptoms among adolescents. The CBCL comprises eight narrow-band scales (Anxious/Depressed, Withdrawn/Depressed, Somatic Complaints, Social Problems, Thought Problems, Attention Problems, Rule-Breaking Behavior, and Aggressive Behavior) and two broad-band scales (Internalizing and Externalizing), and has demonstrated moderate internal consistency and good test-retest reliability (Albores-Gallo et al., 2007). For the present analysis, only *T* scores for the internalizing and externalizing scales were used. *T* scores above 70 were considered clinically significant. For the present study, the Cronbach’s alphas were 0.88 for the internalizing scale and 0.81 for the externalizing scale. Data were obtained from one parent (usually the mother) in both SUD and MCC groups.

### Design and procedure

In the SUD group, instruments were self-administered under the supervision of trained staff (with Master’s or doctoral degrees in clinical psychology) within a month of the patient’s referral to the Addictive Behaviors Unit. In the control group, questionnaires were self-administered in groups on the premises of each participating school, under the supervision of a clinical psychologist with a Master’s degree. The study was explained in detail to parents and participants who gave written, informed consent before entering the study. The evaluation protocol was reviewed and approved by the hospital ethics committee (Ethics committee’s reference number: 5098).

### Statistical analysis

The Student’s *t* test was applied to calculate differences in age between the cases and controls. Analysis of covariance (ANCOVA) was used to identify differences in quantitative variables between groups, with age and number of comorbid diagnoses as covariates. The Chi-squared test and the Fisher exact test were used to compare categorical variables. In addition, effect sizes were calculated for the differences between groups. Bivariate partial correlation coefficients were calculated for age at onset of drug use, level of drug use, internalizing/externalizing psychopathology, and scores on the PSY-5 scales and their facets. Each PSY-5 scale was controlled by the other four remaining PSY-5 scales. We have also performed this analysis for each facet controlling the other remaining facets. Finally, binary logistic regression was used to identify the PSY-5 scales and the facets that best predicted membership of a given group.

## Results

### Descriptive statistics of both groups

Seventy-four SUD cases were included in the final analyses. All met the criteria for SUD according DSM-IV criteria and completed the assessment protocol (mean age, 16.4 years, standard deviation [SD], 0.85, range 15–18 years; 53% male). Eleven percent of participants show a SUD without any comorbid diagnosis. Most participants (89%) had one or more comorbid clinical disorder, including conduct disorder or oppositional defiant disorder (40.5%; *n* = 30), attention deficit hyperactivity disorder (17.6%; *n* = 13), eating disorder (14.8%; *n* = 11), adjustment disorder (12.1%; *n* = 9), non-affective psychotic disorder (10.8%; *n* = 8), anxiety disorder (9.4%; *n* = 7), and mood disorder (6.8%; *n* = 5). In terms of Axis II disorders, 16 (21.6%) adolescents met the criteria for at least one personality disorder. Note that the total percentage is more than 100%, due to cases with multiple diagnoses.

The MCC group comprised 74 adolescents (mean age, 16.0 years [SD = 0.91], range 15–18 years; 53% male) extracted from a representative community group (*n* = 117). MCC were matched with SUD patients by gender. There were no significant differences between the initial community sample and the MCC group with respect to the PSY-5 scales and their facets, behavioral or emotional symptoms, age at first drug use, or patterns of levels of use. The MCC group was therefore representative of the overall community group.

### Differences between SUD cases and controls in PSY-5

Table 1 shows the sociodemographic and clinical data for the SUD and MCC groups, as well as the differences between them. The ANCOVA took age and comorbidity as covariates. The raw score on each of the PSY-5 scales differed significantly between groups, with the score on the Disconstraint scale showing the greatest difference and a large effect size. Almost all the facets of PSY-5 scales also showed significant between-group differences, with the exception of the Grandiosity/Indignation and Low Sociability facets. The two groups also differed in the *T* scores for internalizing and externalizing psychopathology in the CBCL, as well as in the percentages for different levels of substance use (tobacco, alcohol, cannabis, and other drugs). Age at onset of substance use was significantly earlier in the SUD group, although the effect size was moderate-low.

**Table 1:** Descriptive and differences between groups with respect to Personality Psychopathology Five scales and facets, internalizing/externalizing symptoms, age at onset, and level of use for each drug.

Variables	SUD	MCC		
Socio-demographic and clinical variables	(***n*** = 74)	(***n*** = 74)	SUD vs. MCC *t*(*p*)	Effect size
Sex: **n boys/n girls**	39/35	39/35		
Age^a^ **M (SD)**	16.38 (0.85)	16.00 (0.91)	−2.611 (.010)^*^	
**PSY-5 scales** ^1^	**M (SD)**	**M (SD)**	**F (*p*)**	${\boldsymbol{h}}_{\boldsymbol{p}}^{\mathbf{2}}$
Aggressiveness^c^	9.26 (3.97)	6.84 (3.59)	6.16 (.001)^**^	0.11
Psychoticism^c^	3.34 (2.60)	1.81 (2.11)	5.17 (.002)^**^	0.10
Disconstraint^c^	12.77 (4.52)	6.46 (3.48)	30.72 (<.001)^***^	**0.39**
Introversion^c^	7.65 (4.04)	5.84 (4.26)	4.09 (.008)^**^	0.08
Negative Emotionality/Neuroticism^c^	12.05 (4.46)	10.24 (3.90)	4.03 (.009)^**^	0.08
**Facets** ^1^				
Hostility^c^	6.27 (3.02)	3.70 (2.77)	9.98 (<.001)^***^	**0.17**
Grandiosity/Indignation^c^	2.99 (1.50)	3.14 (1.63)	2.22 (.089)	0.04
Psychotic Beliefs/Experiences^c^	1.69 (1.73)	0.69 (1.40)	5.19 (.002)^**^	0.10
Odd Mentation^c^	1.65 (1.23)	1.12 (1.08)	3.62 (.015)^*^	0.07
Delinquent Attitudes^c^	9.36 (3.34)	4.58 (2.43)	33.25 (<.001)^***^	**0.41**
Norm Violation^c^	3.41 (1.63)	1.88 (1.39)	13.67 (<.001)^***^	**0.22**
Low Drive/Expectations^c^	4.39 (2.61)	2.85 (2.00)	7.65 (<.001)^***^	0.13
Low Sociability^c^	3.32 (2.10)	2.99 (2.95)	0.72 (.540)	0.02
**CBCL symptoms** ^2^				
Internalizing^c^	64.64 (14.43)	51.18 (9.69)	15.04 (<.001)^***^	**0.27**
Externalizing^c^	65.58 (10.35)	50.18 (8.52)	28.72 (<.001)^***^	**0.39**
**Age at onset**				
Tobacco^c^	13.48 (1.11)	13.97 (1.44)	1.36 (.261)	0.04
Alcohol^c^	13.81 (0.89)	14.38 (1.24)	3.78 (.012)^*^	0.08
Cannabis^c^	13.86 (1.30)	14.74 (0.96)	3.24 (.026)^*^	0.09
Other drugs (cocaine, amphetamines, etc.)^c^	15.43 (1.17)	16.33 (0.57)	4.80 (.008)^**^	0.13
**Drug use level**				
Tobacco	***N*(%)**	***N*(%)**	**χ^2^(*p*)**	**ϕ**
No Use^b^	0 (0%)	42 (57%)	57.74 (<.001)^***^	**1.17**
Use without problems^b^	12 (17%)	21 (28%)	1.882 (.170)	0.23
Risky substance use^b^	62 (83%)	11 (15%)	35.63 (<.001)^***^	**0.69**
Alcohol				
No Use^b^	3 (4%)	27 (37%)	19.20 (<.001)^***^	**0.80**
Use without problems^b^	34 (46%)	41 (55%)	0.653 (.419)	0.09
Risky substance use^b^	37 (50%)	6 (8%)	22.35 (<.001)^***^	**0.72**
Cannabis				
No Use^b^	0 (0%)	54 (73%)	84.32(<.001)^***^	**1.24**
Use without problems^b^	14 (19%)	18 (24%)	0.50 (.480)	0.12
Risky substance use^b^	60 (81%)	2 (3%)	54.26 (<.001)^***^	**0.93**
Other drugs (cocaine, amphetamines, etc.)	***N*(%)**	***N*(%)**	**χ^2^(*p*)**	**ϕ**
No Use^b^	38 (52%)	72 (97%)	10.56 (.001)^**^	0.30
Use without problems^b^	13 (18%)	2 (3%)	8.07 (.005)^**^	**0.73**
Risky substance use^b^	23 (30%)	0 (0%)	24.73(<.001)^***^	**1.03**

**Notes.**

* *p* < .05.

** *p* < .010.

*** *p* < .001.

a Student’s *t*-test (*df* = 146).

b Chi-squared and Fisher’s exact test (*df* = 1).

c ANCOVA after controlling for age and comorbidity (*df* = 3).

1 Raw scores.

2 *T* scores.

Data in bold face indicate a moderate-large effect size ${h}_{p}^{2}> 0.14$ and *ϕ* > 0.50.

PSY-5 Personality Psychopathology Five CBCL Child Behavior Checklist SUD Substance Use Disorders MCC Matched community control SUP substance use problems

**Figure 1:**
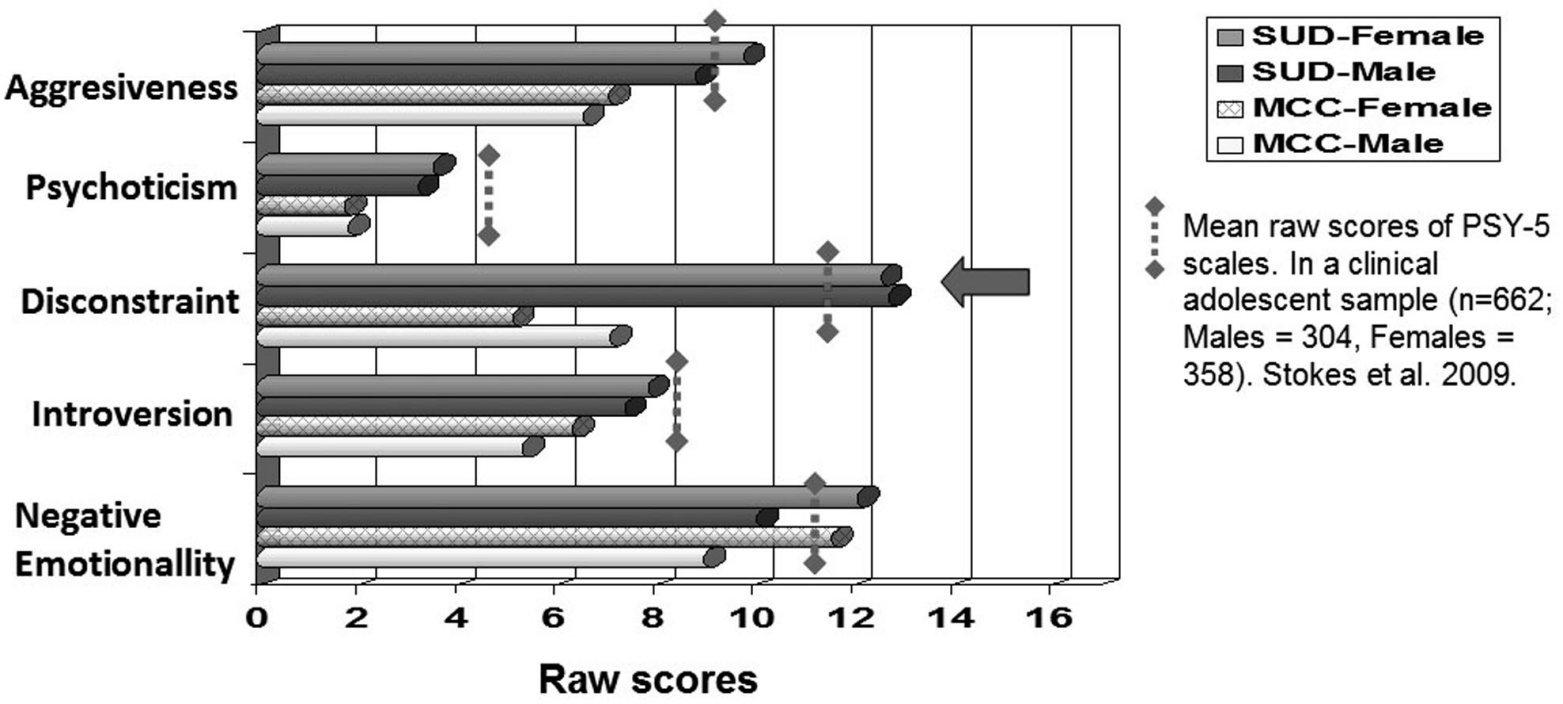
Dimensional raw scores of PSY-5 scales according to gender and type of group.

Figure 1 shows differences among the PSY-5 scales between groups (MCC vs. SUD) by gender. The gray discontinuous threshold represents the mean raw score for PSY-5 scales found in a previous clinical study with adolescents (Stokes et al., 2009). In the present study, gender differences were only found on the Negative Emotionality scale, with females scoring higher in both groups (*p* < .004 for MCC and *p* < .001 for SUD). However, no significant interaction (*F* = 3.016; *p* = .080) was found between gender and group. The largest between-group gender difference was found with the Disconstraint scale (*p* < .001 for both genders).

### PSY-5 and age at onset of drug use

In Table 2, the MCC group showed significant partial correlations for age at onset of alcohol use related to scores on the Discontraint scale, and significant correlations for age at onset of cannabis drug use, mainly associated with the Delinquent attitudes facet. The analysis for the SUD group revealed only one significant correlation between Disconstraint scale and early age of cannabis use. The Delinquent Attitudes facet was associated with an early onset of tobacco, cannabis and other drug use. Also Hostility and Low Sociability facets were associated with early onset of other type of drug use.

**Table 2:** Partial correlations in the matched community control group and Substance Use Disorder group for PSY-5 scales and facets with respect to age at onset, level of drug use, and symptoms (controlling personality scales or facets, respectively).

**PSY-5 scales and facets**		Age at onset				Drug use level				Symptoms	
		TOB	AH	CAN	OD	TOB	AH	CAN	OD	INT	EXT
Aggressiveness^1^	MCC	−.01	.16	.14	–	.15	.11	.11	.18	.03	.10
	SUD	−.01	.08	.17	.38	−.01	−.03	−.07	.01	−.03	.02
Psychoticism^1^	MCC	.03	−.16	−.21	–	−.08	−.06	−.11	−.18	.10	−.01
	SUD	.09	−.06	.21	−.04	.06	.00	.04	−.07	−.04	−.14
Disconstraint^1^	MCC	−.25	**−.31** ^*^	−.33	–	.17	**.35** ^**^	**.44** ^***^	.13	−.10	.08
	SUD	−.18	−.03	**−.29** ^*^	−.16	.19	**.28** ^*^	.22	.19	−.08	.05
Introversion^1^	MCC	−.26	−.14	−.16	–	−.06	−.09	−.06	−.05	**.23** ^*^	−.01
	SUD	−.07	.02	−.06	−.21	.02	−.07	.13	−.01	**.37** ^**^	.06
Negative Emotionality^1^	MCC	.26	.05	−.11	–	.15	.11	.01	.11	.08	.15
	SUD	−.03	−.09	−.11	.01	.18	.20	.22	**.27** ^*^	.21	.22
Hostility^2^	MCC	.17	.18	.13	–	**.24** ^*^	.12	.13	**.27** ^*^	−.08	.13
	SUD	−.10	−.03	.09	−.54^**^	−.02	.07	−.09	.03	−.16	.06
Grandiosity/Indignation^2^	MCC	−.08	.06	−.07	–	.09	.15	.11	.10	.11	.00
	SUD	.06	.05	.04	−.26	−.02	−.08	.13	−.06	.17	−.09
Psychotic Beliefs/Experiences^2^	MCC	.19	−.16	−.34	–	−.05	−.12	**.24** ^*^	**.28** ^*^	.16	.08
	SUD	.16	−.10	.21	−.01	.03	−.01	.04	−.08	−.13	−.02
Odd Mentation^2^	MCC	−.08	−.02	.06	–	−.04	.08	.12	.08	.00	−.03
	SUD	−.10	.01	−.01	.12	.11	.07	.05	.14	.15	−.04
Delinquent Attitudes^2^	MCC	.02	−.18	**−.57** ^**^	–	**.25** ^*^	**.33** ^**^	**.40** ^**^	**.25** ^*^	−.16	−.07
	SUD	**−.32** ^*^	−.14	**−.39** ^**^	**−.42** ^*^	−.13	.21	**.32** ^**^	.18	−.05	.06
Norm Violation^2^	MCC	−.35	−.14	.35	–	−.15	−.02	−.01	−.20	.09	.15
	SUD	.20	.14	.16	.15	−.01	.06	−.14	.05	−.09	.17
Low Drive/Expectations^2^	MCC	.17	−.04	−.04	–	**.25** ^*^	**.28** ^*^	.21	.08	**.23** ^*^	.21
	SUD	−.01	.08	−.04	−.05	.21	−.01	.12	**.26** ^*^	**.39** ^**^	.21
Low Sociability^2^	MCC	−.26	−.08	.15	–	−.07	**−.27** ^*^	−.20	−.04	.01	−.21
	SUD	−.06	−.07	−.07	**−.51** ^*^	−.20	−.06	.12	**−.29** ^*^	.16	−.18

**Notes.**

* *p* < .05.

** *p* < .010.

*** *p* < .001.

Data in bold face indicate a significant difference.

1 Bivariate partial correlations; partial coefficients represent relationship between (age, level and symptoms) and PSY-5 scales with remaiming PSY-5 scales as covariates.

2 Bivariate partial correlations; partial coefficients represent relationship between (age, level and symptoms) and PSY-5 facets with remaiming PSY-5 facets as covariates

TOB Tobacco AH Alcohol CAN Cannabis OD Other drugs INT Internalizing EXT Externalizing MCC Matched Community Control group SUD Substance Use Disorder group

Note: in data of Age onset there are different number of subjects (between MCC and SUD groups and inside the different type of substance), for this reason the correlations have different magnitude on their significance.

### PSY-5 and level of drug use

Also in Table 2, inside the MCC group regarding the level of drug use, the Disconstraint scale was related to high level of cannabis and alcohol use. The Hostility facet was related to tobacco and other drugs. Psychotic Beliefs was associated with a high level of cannabis and other drug use. The Delinquent Attitudes facet was related to tobacco, alcohol, cannabis and other drug use. Low Drive/Expectations was associated with tobacco and alcohol use and Low sociability was associated only with the level of alcohol use. In contrast, in SUD group the level of drug use was positively correlated with, the Disconstraint scale for alcohol, and Negative Emotionality scale for other drugs. The Delinquent Attitudes facet was correlated with the level of cannabis. Other drugs level correlates positively with Low Drive/Expectations and negatively with Low Sociability.

### PSY-5 and behavioral or emotional symptoms

Internalizing symptoms scale of the CBCL in both MCC and SUD groups was related to the Introversion scale, specifically with Low Drive/Expectations facet (Table 2). However, externalizing symptoms measured by the CBCL was not associated with any scale or facet of the PSY-5. These results partially validate the phenomenology of the PSY-5 scales with respect to Achenbach’s model of psychopathology symptoms.

### The predictive value of PSY-5 scales for identifying SUD

In the logistic regression models, we took the groups (SUD vs. MCC) as the dependent variable, and the PSY-5 scales (controlling for age) as independent variables. The results in Table 3 correspond to the only personality psychopathology scale that was related to membership of the SUD group, namely Disconstraint (OR = 1.44; *p* < .001). Subsequent analysis of its facets revealed that Delinquent Attitudes explained most of the group membership (OR = 1.73; *p* < .001), and was an even better predictor than the broad Disconstraint scale. We then constructed a binary variable, based on raw cut-offs of ≥14 in males and ≥12 in females, which were indicative of a pathological Disconstraint condition (these scores represent *T* scores over 60, according to the clinical manuals of the MMPI-A in a US sample). This analysis indicated that the pathological Disconstraint condition achieved a higher odds ratio (OR = 24.4; *p* < .001) than the raw scores of either the Disconstraint or the Delinquent Attitudes scales/facets.

**Table 3:** Predictive values of the Disconstraint scale, the Delinquent Attitudes facet, and the pathological Disconstraint condition in relation to membership of the SUD group.

Variable	OR	95% CI	Hit rate
Raw score Disconstraint scale^a^	1.44^***^	1.28–1.61	79.1%
Raw score Delinquent Attitudes facet^b^	1.73^***^	1.44–2.06	79.7%
Pathological Disconstraint condition^c^	24.40^***^	7.03–85.13	74.3%

**Notes.**

CI confidence interval OR Odds Ratio SUD substance use disorder

a For overall adjusted model (age), *χ*^2^ = 71.52, *df* = 2, *p* < 0.001; Nagelkerke *R*^2^ = 0.51.

b For overall adjusted model (age), *χ*^2^ = 78.07, *df* = 2, *p* < 0.001; Nagelkerke *R*^2^ = 0.55.

c For overall adjusted model (age), *χ*^2^ = 51.70, *df* = 2, *p* < 0.001; Nagelkerke *R*^2^ = 0.39.

* *p* < .050.

** *p* < .010.

*** *p* ≤ .001.

## Discussion

This study is the first to analyze the PSY-5 model of personality psychopathology in a sample of Spanish adolescents that included clinical outpatients with SUD and matched community controls. Significant differences between groups (SUD vs. MCC) were observed on all the PSY-5 scales and on many of their facets, as well as in relation to internalizing and externalizing symptoms and several levels of substance use.

The relationship between PSY-5 personality traits (adolescent information), and internalizing or externalizing symptoms of the CBCL (parent information), showed some of the expected patterns in both groups. Internalizing symptoms were associated with the Introversion scale specifically with Low Drive/Expectations facet. Paradoxically, the expected relationship regarding externalizing symptoms and PSY-5 scales Disconstraint and Aggressiveness were not supported neither in SUD nor in MCC group.

Our results partially confirm previous findings with regard to psychometric properties when applying the PSY-5 model to both adults (Bagby et al., 2008) and adolescents (Bolinskey et al., 2004) or relationships with internalizing symptoms in relation to Introversion PSY-5 scale (Veltri et al., 2009). We also partially replicated the observation that adolescents with SUD score higher on all PSY-5 scales and on many of their facets (Stokes et al., 2009). Additionally, in the MCC group we observed a stronger relationship between the Disconstraint scale and an earlier and more intense use of alcohol and only a more intense use of cannabis. However, in the SUD sample, the Disconstraint is associated with higher use of alcohol and earlier age of onset of cannabis. On the other hand, the Negative Emotionality scale showed some interesting associations mainly with higher use of other type of drugs, which highlights the importance of internalizing symptoms (Hussong et al., 2011), and negative emotionality (Elkins et al., 2006) in the development of SUD in adolescents. Previous studies have reported a high prevalence of psychiatric comorbidity among adolescent patients with SUD (Couwenbergh et al., 2006; Hawkins, 2009; Magallón-Neri et al., 2012), mainly in combination with conduct disorder and other disruptive behavior disorders (Hawkins, 2009), but it is also relevant with affective disorders, specially in girls, as suggested by Díaz et al. (2011).

It is important to obtain evidence for the external validity of the PSY-5 adolescent scales. In particular, it is important to discriminate disruptive behaviors between the Disconstraint scale and other scales that assess externalizing symptoms, drug use, and sexual acting-out as rated through clinical records (McNulty et al., 1997; Stokes et al., 2009). In our study, some relationships are moderately sustained, such as those for early age at first alcohol use, and higher tobacco, alcohol, and cannabis use in the MCC group. In the community group, the Disconstraint scale could be an indicator of risk for the level of drug use. Two findings merit particular attention here. First, the Delinquent Attitudes facet of the Disconstraint scale was associated with the level of any substance use in MCC group and specifically with cannabis in SUD group. Second, the Negative Emotionality scale was related to the level of use for other drugs.

In regard to substance use onset, some interesting relations with the PSY-5 scales emerge. For instance, one of our hypotheses, i.e., that early substance onset may be related to the Disconstraint scale, is partially ratified, especially in regard to early alcohol use in our non-clinical sample, as previous studies have shown (Chassin, Pitts & Prost, 2002; Iacono, Malone & McGue, 2008; Tarter et al., 2003), and early cannabis use in SUD group. The Disconstraint facet Delinquent Attitudes was associated with an early onset of cannabis use in the MCC group and early onset of tobacco and cannabis and other drug use in the SUD group. Although we expected clearer signs of the association of the Disconstraint scale with early substance use, the evidence is that Delinquent Attitudes is the most specific facet in the PSY-5 model for detecting problems related to early substance use in adolescence. Also, it is relevant to remark that Hostility and Low Sociability are related with early onset of other type of drugs. On the other hand, Negative Emotionality seems to be related to substance use associated with a component of emotional dysregulation, psychological discomfort and the self-medication mechanism for mitigation, escape or coping with problems related to this discomfort (Baker et al., 2004; Hawkins, 2009). Due to the early onset of substance use, it is associated with a more severe course of illness and higher odds of diverse risky behaviors (Castellanos-Ryan et al., 2014; Malmberg et al., 2010). Some brief school-based coping skills interventions targeting personality risk factors for adolescent substance use have been shown delay onset or early substance use (Castellanos-Ryan & Conrod, 2011).

In regard to the level of substance use, it should be noted that the Delinquent Attitudes facet was a consistent and specific indicator of higher usage levels in both groups. In the control group, it indicated levels of tobacco, alcohol, cannabis use and other drugs, and in the SUD group it indicated higher cannabis use. This is important because, although the Disconstraint domain explained a considerable proportion of the variance in problems associated with drug use, and the pathological condition of this scale indicated a higher risk of belonging to the SUD group, the Delinquent Attitudes facet may explain much of the apparent relationship. A more in-depth study of this and other facets (i.e Low Drive Expectations and Low Sociability) is therefore required to understand the true impact of these behaviors in adolescents with SUD. This could help the future development of preventive interventions for the adolescent community population that are based on personality profiles and coping strategies (Castellanos-Ryan, O’Leary-Barrett & Conrod, 2013). These results support the use of PSY-5 to identify risk cases for SUD, as well as to plan specific treatments tailored to individual personality styles of the patients (Hawkins, 2009; Hicks, Iacono & McGue, 2014; Magallón-Neri et al., 2012).

Although the MMPI-A is widely used inside clinical and forensic settings (Archer, 2005; Stokes et al., 2009; Veltri et al., 2014) and can help to obtain indicators of general psychopathological disturbances in clinical patients (such as SUD) and community samples (Bolinskey et al., 2004), due to its length it can be difficult to apply in certain contexts, specially if patient motivation is low. For this reason, it is important to develop shorter instruments in order to extract the essence of the PSY-5 model, without forgetting the indicators of test internal reliability (L, F and K scales) which enable clinicians to discern between profiles with some degree of simulation (fake-bad or fake-good). In the present study, the PSY-5 model shows potential for developing screening or phenotypic risk markers indicators associated with substance use abuse or dependence in adolescents, especially if scores on the Disconstraint scale are high. Other scales i.e SURPS (Castellanos-Ryan & Conrod, 2011; Woicik et al., 2009), specifically in a Spanish version (Robles-García et al., 2014) may become important tools for daily clinical and research practice in the applicability to identify personality pathology characteristics associated with young people at risk for developing substance use problems.

### Limitations and strengths

Several limitations should be considered when interpreting these results. First, we mostly analyzed self-reported measures to assess psychopathology in the control group. However, these types of instruments guaranteed confidentiality and contributed to the reliability results when assessing psychopathology, personality and substance use in adolescents. A more detailed assessment via structured clinical interviews would be preferable for the analysis of personality psychopathology because, depending on the type and methodology used for detection, the descriptions can lead to over- or under- estimations of their impact (Magallón-Neri et al., 2014). Second, although the comparison groups were matched for gender, it would have been desirable to consider more sociodemographic variables. However, the study group characteristics did not allow this, and the influence of both gender, age and number of comorbid diagnoses were controlled. The third limitation is the lack of Spanish scales for the PSY-5 dimensional model. The fourth limitation is related to categorization in some variables and the limited use of sophisticated SEM analyses due to restricted sample. Additionally, the transversal design of the study prevents the possibility of analyzing if comorbid conditions are the cause or the consequence of substance use related problems.This is an issue that is worth analyzing in future studies. Despite these limitations, the current study has a number of strengths that have been highlighted; these aspects that can be considered innovatory in our study or not treated in previous studies. Some scales such as Disconstraint, and specifically the facet of delinquent attitudes, we found to be useful for the screening of problems associated with SUD in adolescents. In addition, this study is one of the few to use matched clinical and control groups instead of exclusively concentrating on adolescent alcohol use. We also focused on tobacco, cannabis and other drugs. Finally, a considerable strength of the study is that, to our knowledge, this is the first study in a Spanish or Latino context in use the personality psychopathology (PSY-5) model in adolescents to identify substance use risk, which is very close to the new domains of the alternative DSM-5 model of personality pathology.

## Conclusions

Although the present results are preliminary and need to be replicated in larger samples, this study illustrates how a dimensional approach to personality pathology can be helpful in the assessment of adolescents with respect to the risk for development of substance use problems. This highlights the importance of careful and individualized assessments of personality in adolescence.

## Supplemental Information

10.7717/peerj.992/supp-1 Supplemental Information 1 Dataset PSY-5 Dataset converted SPSS to CSV
